# Authors’ Reply – Comments: Influence of the prone position on a stretcher for pregnant women on maternal and fetal hemodynamic parameters and comfort in pregnancy

**DOI:** 10.6061/clinics/2019/e958

**Published:** 2019-04-16

**Authors:** Claudia Oliveira, Marco Antonio Borges Lopes, Agatha Sacramento Rodrigues, Marcelo Zugaib, Rossana Pulcineli Vieira Francisco

**Affiliations:** I Fisioterapia, Universidade Santa Cecilia, Santos, SP, BR; II Departamento de Obstetricia e Ginecologia, Faculdade de Medicina FMUSP, Universidade de Sao Paulo, Sao Paulo, SP, BR; III Instituto de Matematica e Estatistica, Universidade de Sao Paulo, Sao Paulo, SP, BR

We would like to thank Heazell AEP et al. 1 for their interest in our study 2.

In fact, to our surprise, in our study, we found that there were no signs of a decrease in cardiac output in the supine position as observed in traditional stretchers according to the studies in the literature.

We believe that there were no significant hemodynamic changes and that the parameters remained within normal levels when the pregnant women were in the supine position on the special stretcher for up to 6 minutes for two reasons. First, reduced compression of large vessels occurs; this is due to the slight 15-degree elevation of the dorsal region of the woman's body with a pillow to support her lumbar spine and to the shape of the stretcher, which contains an opening that creates a space to accommodate and support her pelvic region, thus anatomically maintaining the curve of her lumbar spine. Second, pain occurs when women are lying on traditional stretchers, which can lead to changes in hemodynamic parameters due to the stress this position creates; however, the women in our study indicated no pain or discomfort in the supine position.

Regarding safety in the prone position, Nakai et al. 3 describe in their study that the inferior vena cava is situated on the right side of the spine and may continue to be compressed by the uterus due to the flexibility of the gravid uterus when a pregnant woman is in the left lateral decubitus position. However, the authors report that the prone position is the only one that completely decompresses the large vessels, which makes the prone position safe. We therefore conclude that the uterine compression of the large vessels is not completely relieved in the left lateral position and only the maternal prone position could provide complete relief of uterine compression.

We would like to note that all pregnant women in both groups in our study were in the prone position twice and that their blood pressures decreased, which allowed us to affirm that this position was safe during the 6 minutes of evaluation on the special stretcher.

As we mentioned in the discussion of our study, we agree that further research is necessary to analyze the effects of the prone position on the special stretcher for longer than six minutes, such as for the total duration of a physical therapy session or medical procedure. We are certainly striving to carry out such research. We appreciate the words of encouragement.

## References

[1] Heazell AEP, Stone P (2019). Comments: Influence of the prone position on a stretcher for pregnant women on maternal and fetal hemodynamic parameters and comfort in pregnancy. Clinics.

[2] Oliveira C, Lopes MAB, Rodrigues AS, Zugaib M, Francisco RPV (2017). Influence of the prone position on a stretcher for pregnant women on maternal and fetal hemodynamic parameters and comfort in pregnancy. Clinics.

[3] Nakai Y, Mine M, Nishio J, Maeda T, Imanaka M, Ogita S (1998). Effects of maternal prone position of the umbilical arterial flow. Acta Obstet Gynecol Scand.

